# Digital Mental Health for Young People: A Scoping Review of Ethical Promises and Challenges

**DOI:** 10.3389/fdgth.2021.697072

**Published:** 2021-09-06

**Authors:** Blanche Wies, Constantin Landers, Marcello Ienca

**Affiliations:** Department of Health Sciences and Technology, ETH Zurich (Swiss Federal Institut of Technology), Zurich, Switzerland

**Keywords:** ethics, digital health, mental health, adolescent and youth, digital health (eHealth)

## Abstract

Mental health disorders are complex disorders of the nervous system characterized by a behavioral or mental pattern that causes significant distress or impairment of personal functioning. Mental illness is of particular concern for younger people. The WHO estimates that around 20% of the world's children and adolescents have a mental health condition, a rate that is almost double compared to the general population. One approach toward mitigating the medical and socio-economic effects of mental health disorders is leveraging the power of digital health technology to deploy assistive, preventative, and therapeutic solutions for people in need. We define “digital mental health” as any application of digital health technology for mental health assessment, support, prevention, and treatment. However, there is only limited evidence that digital mental health tools can be successfully implemented in clinical settings. Authors have pointed to a lack of technical and medical standards for digital mental health apps, personalized neurotechnology, and assistive cognitive technology as a possible cause of suboptimal adoption and implementation in the clinical setting. Further, ethical concerns have been raised related to insufficient effectiveness, lack of adequate clinical validation, and user-centered design as well as data privacy vulnerabilities of current digital mental health products. The aim of this paper is to report on a scoping review we conducted to capture and synthesize the growing literature on the promises and ethical challenges of digital mental health for young people aged 0–25. This review seeks to survey the scope and focus of the relevant literature, identify major benefits and opportunities of ethical significance (e.g., reducing suffering and improving well-being), and provide a comprehensive mapping of the emerging ethical challenges. Our findings provide a comprehensive synthesis of the current literature and offer a detailed informative basis for any stakeholder involved in the development, deployment, and management of ethically-aligned digital mental health solutions for young people.

## Introduction

Mental health disorders are complex disorders of the nervous system characterized by a behavioral or mental pattern that causes significant distress or impairment of personal functioning (1). These include, among others, anxiety, depression, substance use disorders, schizophrenia, eating disorders, bipolar disorder, and post-traumatic stress disorder. Mental health disorders compose a significant portion of the global burden of disease. In 2017, 970 million people worldwide had a mental health disorder, comprising approximately 13% of the global population. Since then, it is estimated that mental health conditions have increased worldwide as they now cause on average 1 in 5 years lived with disability (2). The mortality rate of people with mental disorders is significantly higher than the average population, with a median life expectancy loss of 10.1 years. Mental health disorders are attributable to eight million deaths each ear, that is 14.3% of deaths worldwide (3).

Mental illness is of particular concern for younger people. The WHO estimates that around 20% of the world's children and adolescents have a mental health condition, a rate that is almost double compared to the general population. Mental-illness-induced suicide is the second leading cause of death among 15 to 29-year-olds. Despite these figures, the global median of government health expenditure that goes to mental health is <2% (2). To make things worse, the epidemiology of mental illness is expected to be exacerbated by the ongoing new Coronavirus disease 2019 (COVID-19) pandemic. A recent survey has found that the pandemic has affected the mental health of 59% of people in the United States (4). Research shows that the ongoing COVID-19 pandemic is contributing to widespread emotional distress and increased risk for psychiatric illness, either directly associated with the COVID-19 illness or indirectly through imposition of restrictive public health measures that infringe on personal freedoms and associated financial losses (5). Furthermore, people with serious mental illness have been observed to be disproportionately affected by the pandemic (6). This impact has been particularly disruptive for young people, many of whom have self-reported increased mental health issues as a result of lockdowns. A recent survey conducted among 13–25 years olds with a history of mental health needs in the United Kingdom found that 67% of respondents believe that the pandemic will have a long-term impact on their mental health (7).

One approach toward mitigating the medical and socio-economic effects of mental illness is leveraging the power of digital health technology to deploy assistive, preventative, and therapeutic solutions for people in need. As a consequence, digital mental health is a growing field of interest in digital health and scientific research. We define “digital mental health” as any application of digital health technology for mental health assessment, support, prevention, and treatment. This technological cluster includes mobile health (mHealth) applications, wearables, consumer neurotechnologies, virtual reality systems, online platforms, care coordination systems, assisted living ecosystems etc.

Young people are the primary end-users or patient groups of digital mental health tools: they are early adopters of all things digital, including digital health (8). The relevance of leveraging digital mental health solutions has further increased as a consequence of the COVID-19 pandemic because of both the increased prevalence of mental illness and the growing demand of telemedicine services (9). The application of digital health methodologies to young people thus promises considerable benefits and has received growing attention in the literature. However, this age group is also particularly vulnerable and susceptible to manipulation, especially via digital devices and methods. As a result, the use of digital technologies for mental health treatment among adolescents and children generates benefits and ethical issues.

Growing evidence suggests that digital mental health can improve mental health conditions such as depression across various patient populations (8, 10, 11). However, there is only limited evidence that digital mental health tools can be successfully implemented in clinical settings (12). Authors have pointed to a lack of technical and medical standards for digital mental health apps (13), personalized neurotechnology (14) and assistive technology for age-related cognitive decline (15) as a possible cause of suboptimal adoption and implementation in the clinical setting, Mohr et al. (12) have suggested that digital mental health research should therefore be solution-focused to develop pragmatic solutions. Further, ethical concerns have been raised related to insufficient effectiveness (14), lack of adequate clinical validation and user-centered design (16) as well as data privacy vulnerabilities (15) of current digital mental health products. Assessing the benefits and risks of digital mental health systems requires, therefore, a careful balancing act and a holistic approach to scrutinizing the advantages that these socio-technical trends can bring for mental health patients while minimizing their unintended risks. Most importantly, it requires a careful risk-benefit analysis that could inform ethical guidelines, policy interventions, oversight mechanisms and clinical decision making in this domain.

The aim of this paper is to report on a scoping review we conducted to capture and synthesize the growing literature on the promises and ethical challenges of digital mental health for young people. We define “young people” as the combined group of children and adolescents—concretely people in the age group from 0 to 25 years. This review seeks to survey the scope and focus of the relevant literature, identify major benefits and opportunities, and provide a comprehensive mapping of the emerging ethical challenges. Our findings provide a comprehensive synthesis of the current literature and offer a detailed informative basis for any stakeholder involved in the development, deployment and management of ethically-aligned digital mental health solutions for young people.

## Methods

The objective of this review was to gather information about the benefits and ethical challenges regarding digital technologies for mental health treatment and assessment among adolescents or children. To this purpose, on the 7th of October 2020 we searched five databases (PubMed, Scopus, World of Science, PsychInfo, IEEE Xplore, and the ACM Digital Library) in order to retrieve eligible publications. The following search string was used:*((“social media” OR “Digital Media” OR “big data” OR “Artificial Intelligence” OR “digital phenotyping” OR “digital mental health” OR “digital biomarkers” OR “mental health apps” OR “digital sensors” OR “digital mental health technologies” OR “health related Apps” OR “mobile Health” OR eHealth OR smartphones OR wearables OR “Holter monitoring”) AND (ethics OR bioethics OR “bioethical issues” OR “ethical issues” OR “ethical analysis” OR “ethical review”) AND (“mental health” OR “mental well-being” OR “emotional health” OR “emotional well-being”) AND (“young adult” OR young OR adolescent OR child OR teenager))*.

**Figure 1:**
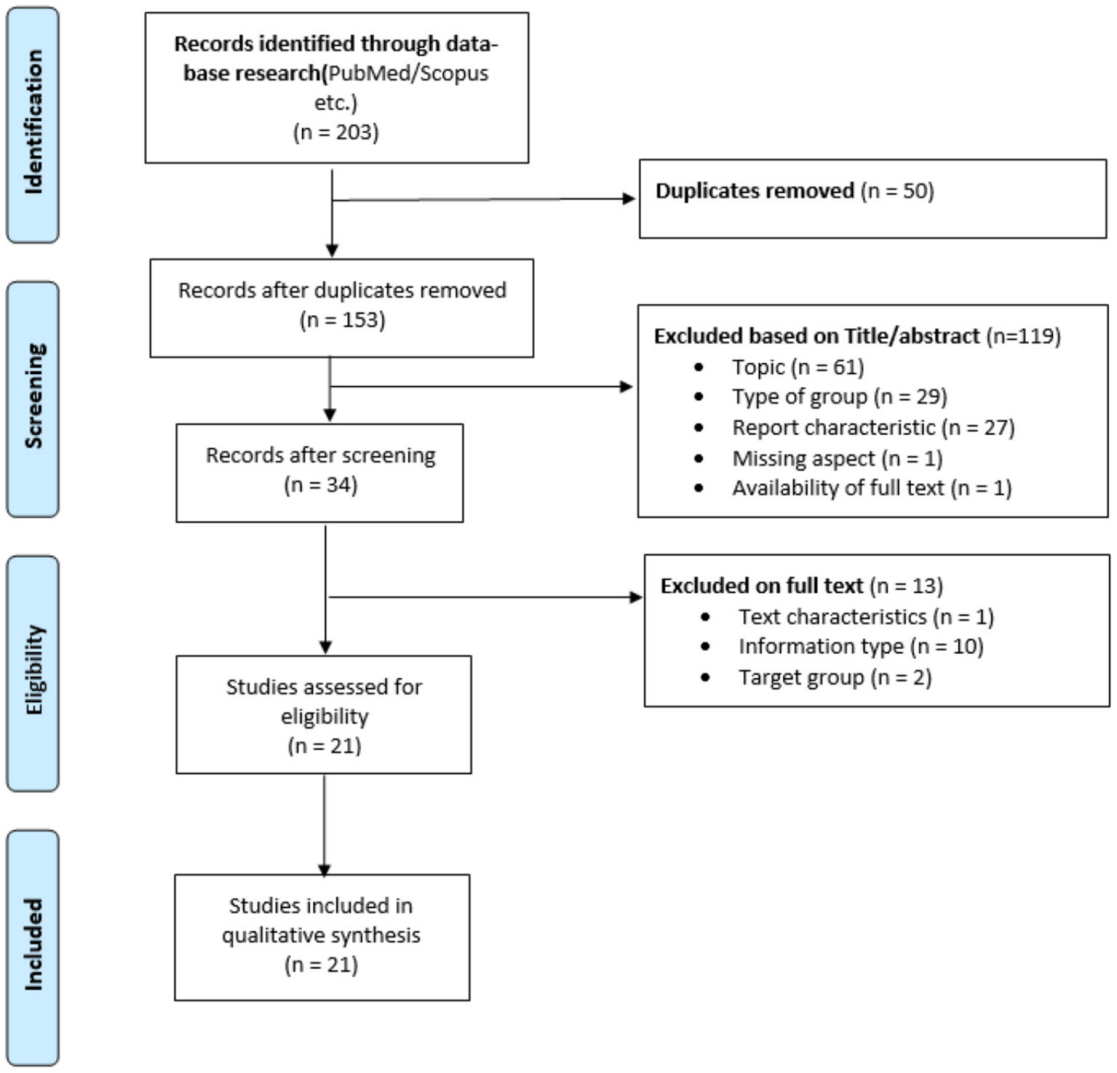
PRISMA flow chart.

Based on the PRISMA Statement and flowchart, four phases of review were conducted: identification, screening, eligibility assessment and final synthesis (see Figure 1). Our search string initially retrieved 203 papers. All entries were exported into the Endnote reference management software. Automatic duplicate removal was performed. Fifty articles were identified as duplicates and therefore removed. The remaining 135 articles were screened based on Title/Abstract and assessed based on the inclusion and exclusion criteria (see Annex 1 in Supplementary Material). Thirty-four articles passed the eligibility assessment and were included into the final synthesis. Articles were deemed eligible if they suited the following inclusion criteria: (a) original peer-review journal publication; (b) written in English, German, Spanish, Italian, or French (languages spoken by the research team); (c) published between 2015 and October 2020; (d) describing/assessing ethical considerations relating to digital health technologies designed for or utilized by children and/or young adults (under 25) for mental health support or otherwise related to the promotion of mental health.

In addition to this systematic review component and compatibly with the best practices for scoping reviews, we conducted a grey literature analysis via non-academic search engines and citation chaining. To this purpose, we used multiple unstructured combinations of the search string. This led to the inclusion of 9 additional articles to the final synthesis.

A total of 26 articles were included into the final synthesis and an in-depth review of full-text articles included in the synthesis was performed. Data were analyzed through qualitative thematic analysis with assistance of the MAXQDA data analysis software. Through the establishment of a keyword coding system, recurrent thematic patterns were inductively identified and subsequently grouped into different themes and subthemes. Our analysis consisted of three sequential steps. First, for each article, we screened the presence of ethically relevant considerations. During this phase, ethically relevant keywords and statements were searched in the full texts of all reviewed articles. This process was performed by two authors using both software-guided keyword search (software used: Endnote X9) and unguided full-text review. Second, we clustered all retrieved ethical considerations into main thematic families using thematically oriented content analysis (17). Each thematic family was further classified into sub-families relative to specific sub-components of the main ethical theme. When the same digital health technology description contained more than one ethical consideration, all considerations were allocated to their respective thematic families and subfamilies.

## Results

**Figure 2:**
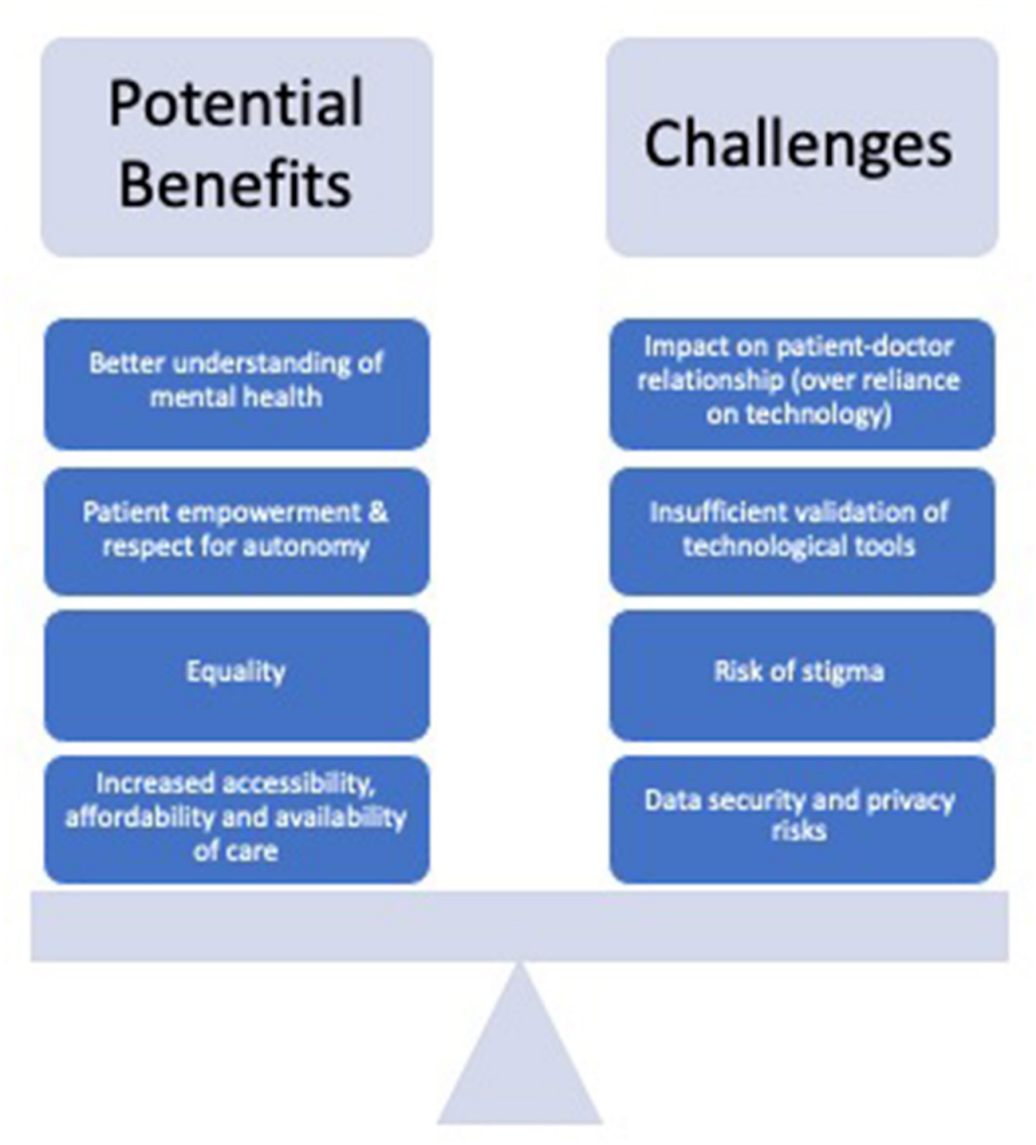
Expected benefits and ethical challenges related to digital mental health for young people.

The analysis showed a diverse range of themes relating to the opportunities and ethical challenges of using digital mental health technologies among young people. Figure 2 provides a visual overview of key codes and themes that emerged from our analysis.

### Ethically Significant Benefits and Opportunities

#### Accessibility

The most recurrent expected benefit associated with the use of digital mental health is the prospect of increased accessibility to health care (18–21). This assessment was based on the increased affordability of mental health apps or internet-based platforms in comparison to face-to-face consultations (22–27) and the easy access given the liberation of geographical restraints (22–25, 28–30). This potential benefit is of ethical significance because increased access to healthcare is a critical to promote health equality and justice. Furthermore, digital mental health solutions were expected to provide more continuous, around-the-clock availability of help or support (18, 23–26, 29, 31, 32). By increasing accessibility, digital mental health technologies were also seen to hold potential for increasing equality between different population groups (21, 24–26, 33, 34), as well as within the patient-therapist relationship (32).

#### Enhanced Therapy Facilitation and Prevention

The usage of digital mental health therapeutics is also seen to facilitate the therapy, prediction and prevention of mental illness of the patient (24, 26, 29, 31) The beneficial potential of digital mental health technology for continuous and accessible care delivery is of particular relevance to low-to-moderate cases that are not being evaluated and treated, especially in areas where mental health care resources are under high levels of pressure due to severe mental illness cases. At the same time, however, our findings underscore that digital mental health, albeit already useful for monitoring purposes, has yet to become effective for predictive purposes. For example, Mulder et al. (35) and Chan et al. (36) cautioned to redirect attention from algorithmic prediction of suicide to a causal pathway and called for paying more attention to real engagement with the individual patient, their specific problems and circumstances. Although machine learning algorithms appeared to improve existing decision support tools, their usefulness in the clinical setting was deemed limited.

#### Autonomy and Empowerment

Another important theme was the potential of digital mental health technologies to increase the autonomy and sense of empowerment of young adults (24, 29, 32). The use of digital mental health tools gives youngsters, on the one hand, the chance to play a more active role in their own treatment as they can actively seek support or control difficult situations (18, 20, 25, 26, 29, 31, 32), as well as the pace of answering/responding (26). Additionally it gives the patient the opportunity to implement the learned coping strategies outside the therapy setting and thereby increases patient autonomy and sense of empowerment (33, 34). On the other hand the technologies fostered autonomy through providing easier access to information and support or more generally, the possibility to manage mental health and well-being (27, 29, 32, 34). Additionally, some authors argued that an increased empowerment leads to higher responsibility for taking care of one's own mental health development, which is an important step in the treatment of mental illness (18).

#### High Acceptability Among Young People

As a substantial part of the youth's social interactions and life take place in the digital space, e.g., through the use of social media, young people's perspectives and choices regarding multiple digital mental health related topics are influenced by the digital ecosystem in which they are embedded. They are more inclined to accept the use of digital tools for the assessment, treatment or support of mental health issues (27). Further young people use the digital space (e.g., the Internet) as a trusted source of information (19, 22, 37) or see it as an easier way to start to talk about mental health, their problems or to seek help (27, 32, 34, 38). In addition, our analysis suggests that the acceptability of using digital mental health technologies may also be positively influenced by their potential for enabling more anonymous interactions compared to face-to-face meetings with health professionals (24, 25, 38). The flexibility and anonymity of the digital space allows young adults to avoid social stigma or exclusion and increases their feeling of comfort to share personal data as you can quickly access the therapy tool through your smartphone (22, 24–27, 38).

Further the type of data that can be collected through mental health apps, chatbots, or social media may well lead to an increased understanding of mental illnesses, as more data is available for analysis (19, 20, 23, 24, 37, 38). The increased amount of data and positive attitude of people toward the usage, could ultimately help to eradicate or at least decrease the stigma that is attached to mental illness (24, 26). Lee et al. further discussed the promise of transferring the trust given to chatbots to professionals (38).

### Disadvantages and Ethical Challenges

#### Privacy and Confidentiality

The most frequently mentioned risks of digital mental health technologies addressed in the literature regard the privacy, confidentiality and security of the user's data and information obtained through digital mental health applications. The biggest concerns expressed by authors regards what happens if confidential information is shared with or access given to third parties (19, 20, 23–27, 31, 38–43). Authors argued that the negative consequences of insufficiently secured data sharing can reach into multiple domains of life, such as work, school or even into relationships with friends, families or partners (19).

#### Patient Mistrust Due to Privacy and Confidentiality Concerns

Mistrust in data sharing due to privacy concerns and confidentiality breaches may reduce the effectiveness of mental health treatments. Authors argued that if the data are insufficiently secured, hence at risk of being breeched, multiple negative consequences are expected to arise from patient mistrust. First, patients' trust in their psychiatrist or psychotherapist may be lost, tarnishing or at least negatively influencing the doctor-patient relationship (19, 26, 28, 32, 34, 41). Second, the prospect of privacy breaches and security vulnerabilities is expected to decrease the acceptability of digital mental health technologies (40, 43), leading to an even bigger vulnerability of already exposed people and increasing the unease and uncertainty of the users toward the technology (19, 38, 40, 41, 43). Other authors highlighted that mistrust in digital mental health technologies is further aggravated by the fact that patients often feel that technologies collect too much information (38) and develop sceptical attitudes due to the rapid speed of technological evolution (21, 38).

#### Pervasive Stigma

It has been observed that digital mental health technologies can increase the risk of stigmatization for young adults and children, especially where relevant data have been exposed (26, 40, 43). Stigmatization may lead to various devastating effects in young peoples' lives. Cyberbullying is widespread and may be particularly burdensome where data is leaked (40). Digital technologies, in particular social-media, may elicit addiction and reinforce self-harming behavior. Internalized stigma may lead patients to use social networks to self-expose such self-harming behavior, which in turn may reinforce stigma against their illnesses (44). The effects of stigmatization extend to how patients are treated by institutions. Feuston and Piper argued that institutional representations of mental illness, such as the media, contribute to stigma by providing “unfavourable and inaccurate representations of psycihatric disorders” (45). Martinez-Martin and Kreitmair have shown that addiction-induced illegal drug use has had legal consequences for patients when digital mental health technology providers had to share personal information (40).

#### Accessibility and Equal Access

Although enhanced access is often seen as a major benefit of digital mental health (19, 20, 34), issues related to accessibility and equal access are nonetheless identified as major ethical concerns (21, 34, 46). Authors observed that systemic issues, such as socio-economic inequality, lead to considerable structural barriers to access (18, 47, 48). One widely referred to barrier is the *digital divide* that describes the phenomenon that technology is not equally available to all social groups due to economic, social or cultural inequalities (21, 24, 49). Concretely, barriers such as poor network coverage in rural areas or the cost of digital communication constrain disadvantaged children and young adults to access relevant content (34). Without digital literacy or adequate access, patients may face severe disadvantages, as they are prevented from accessing novel mental health care solutions (49). Similarly, although digital mental health technologies hold potential for increasing the autonomy and sense of empowerment of young adults (24, 29, 32), they also raise the risk of diminishing patient autonomy by increasing the risk of digital addiction and manipulation.

#### Cross-Cultural and Cross-Country Attitudes and Resources

Cross-cultural and cross-country differences in attitudes and resources have been associated to considerably different standards of care. Sharma showed that stakeholders' socio-technological aspirations to technology for disabled children differ considerably among Indian and Finnish stakeholders (21). This is, in their view, attributable to differences in resource availability and government engagement in “developed” and “emerging” economies. This raises further issues as healthcare systems in which mental health resources are under high pressure often fail to address the needs of patients with less severe illnesses—leading to systemic issues around not serving the underserved (47).

Some researchers and app developers have deliberately attempted to react to these issues by developing products and approaches that enable access to emotional self-regulation and mental health prevention among the socioeconomically disadvantaged (18) or disabled (21). However, Sharma showed that currently available technology is still insufficiently engaging and inclusive to fully offset these concerns (21).

Finally, the absence of regulatory clarity concerning the responsibility for data leaks and potential dignitarian or other digital harms caused by technology misuse, render digital mental health technologies less trustworthy (32, 40, 43).

#### Clinical Validation and the Need for Ethical-Legal Guidance

Other repeatedly discussed topics were the unclear efficacy and effectiveness (25, 27, 30, 31, 33, 34, 40), translational challenges and the uncertain feasibility of successful implementation (25) as well as uncertain reliability (19, 27, 28, 43, 50) of these technologies. Authors emphasized that many mental health apps and internet-based platforms are not subjected to extensive and professional evaluations or clinical validation studies, which leads to unclear influences and outcomes. This uncertainty is exacerbated by the fact that little ethical and regulatory guidance currently exists for digital mental health. To reduce uncertainty and bridge this normative gap, several authors called for the necessity of developing an ethical-legal framework for digital mental health technology, chiefly through ethical guidelines, recommendations, and best practices.

Blurring the boundary within the doctor-patient relationship, increasing the risk of over-reliance on digital mental health technologies as well as poor conduct were also identified as ethically relevant challenges (26, 31, 51). Authors expressed concerns about the unrealistic expectations of around-the-hour-availability of psychiatrists through mails or text messages (32, 37) which could pose additional burden on health professionals. In addition, digital mental health applications could facilitate the sharing of personal information among both parties involved through ill-suited channels (26, 32, 34) and blur the boundaries of a psychiatrist's assessment by including deontologically questionable activities such as checking a patient's social media accounts (26, 32).

#### Consent and Dependency

Another challenge identified in the literature is the unclearly defined expectation of which parties have to consent to data processing in digital mental health applications (19, 26, 37, 41). Authors also reported insufficient clarity about the adequacy of consent obtained through digital mental health applications, in particular regarding the type of data processing or intervention that the user is consenting to (28, 40, 41, 43).

As shown by Lee et al. ensuring data and algorithmic transparency when processing users' personal information is very important (38). The over-reliance and the all-time availability of these technologies are feared to influence the young adults' capabilities of social interactions with the ancillary risk of diminishing their trust to talk about problems with their friends (43) or increase the dependency of young people on clinical support (32). In addition to that, websites and chatbots are often intentionally designed to get the users dependent on the technologies. Notably in young adults this can ultimately increase addictive behaviors (27, 29). Further it can decrease the feeling of responsibility of the young people to take care of their health as they expect that it is done for them (32, 34) and additionally diminish their willingness to attend face-to-face consultations (34).

#### Other Topics

Further topics that were discussed, though with less frequency, in the literature included issues of accountability, liability, anonymity, the relation of digital mental health and human rights as well as the evaluated role of these technologies based on different ethical frameworks such as principlism, ethics of care and utilitarianism. The discussion around accountability and liability was for instance emphasized by Martinez-Martin et al. as they flagged the limited applicability of traditionally defined therapeutic codes to providers of direct-to-consumer (DTC) technologies. The authors observed that the same rules of conduct that prevent malpractice or liability issues in traditional therapy settings are not precisely applicable to digital psychotherapies, especially those administered through DTC software and devices. This creates a problem of sub-optimal accountability for e.g. chatbots to establish a safe and trusting relationship with patients (40).

## Limitations

This study presents four main limitations. First, it may be affected by a selection bias because the search retrieved only articles written in languages known by the researchers (English, Spanish, French, German, and Italian), excluding articles written in other languages. A similar limitation affects database selection: screening additional databases may have possibly identified additional relevant studies. Finally, our study included only peer-reviewed articles in scientific journals, hence excluded other articles sources such as conference proceedings and book chapters. The risk of selection bias is inherent to any review because the number of databases that can be feasibly searched is always finite. We attempted to minimize selection bias by exploring both domain-general (Web of Science, Scopus) and domain-specific databases including the major databases in biomedical research, psychology and computer science. Second, exhaustiveness is not the objective of scoping reviews as the explorative nature and broad focus of this methodology makes it “unrealistic to retrieve and screen all the relevant literature” (52). With regard to article types, although we recognize that including also non-journal articles such as conference proceedings may have the valuable consequence of adding to our synthesis papers from conferences highly competitive discipline specific conferences (especially in computer science), it could thereby have the adverse effect of including low-quality unscrutinised contributions from other domains. Therefore, we considered restricting the synthesis to peer-reviewed journal articles a valid quality control mechanism.

## Discussion

As often observed, mental health is a public health priority. Developmental psychiatry research indicates that most mental health disorders begin in childhood and adolescence. This raises an additional medical and ethical duty to detect and assess mental health needs early and treat them during child development. Therefore, deploying digital solutions that can reliably monitor and identify mental health needs during early phases of psychological development is an inherently ethical task. These technologies hold promise for alleviating the burden of mental illness, reducing the risk that critical health needs during this sensitive time of child development remain undetected, providing novel assistive and therapeutic resources for young people in need and improving practical aspects of mental healthcare delivery. This is particularly valuable since untreated mental health problems originating during childhood and adolescence can reportedly lead to future negative health and social outcomes. At the same time, research in digital ethics has largely shown that digital health devices and software raise a variety of ethical challenges, especially challenges related to privacy, equality of access, patient autonomy. These challenges may be exacerbated when digital health solutions are designed for and accessed by children and adolescents, as young people with chronic mental conditions belong to vulnerable groups and are often below the age of consent for medical treatment. For this reason, deploying digital mental health solutions for young people requires a proactive ethical assessment which carefully balances the benefits that these technologies can bring against the possible collateral risks.

Our thematic analysis shows that increasing accessibility to mental healthcare is a core ethically relevant opportunity enabled by digital mental health. Our findings reveal that the increased affordability of digital mental health tools in comparison to face-to-face consultations combined with limited dependence of these systems on geographical constrains may facilitate access to mental healthcare. Research has shown that the provision of mental health services is currently constrained by structural barriers, with many people facing insufficient access to diagnostics and treatment (47, 48). As a consequence, more than half of adults with mental illness in countries such as the U.S. do not receive mental healthcare treatment. From a public health perspective, the increased affordability of mental health apps and internet-based platforms in comparison to face-to-face consultations is likely to facilitate access to mental healthcare in countries such as Switzerland and the United States where care provision is not entirely reimbursed through public finances. In addition, it is likely to expand access to mental healthcare in World regions such as rural areas and low-and-middle-income countries where institutional care provision is limited. Even in areas where access to care is not constrained, the around-the-clock availability of digital tools holds promise for improving prevention, help and support.

From a patient perspective, the potential of digital mental health technologies to increase the autonomy and sense of empowerment of young adults merits particular attention. In biomedical ethics, the principle of autonomy is typically understood as the capacity of the person to deliberate or act on the basis of one's own desires, that is the ability to act freely in accordance with a self-chosen plan (53). Digital mental health tools hold promise for giving young patients the chance to play a more active role in their own treatment and provide them with the opportunity to actively seek support or control difficult situations. Additionally, they give them the chance to refine coping strategies learned outside the therapy setting and gain easier access to information and support (27, 29, 32, 34). Our findings illustrate that young patients may be more inclined to seek mental health support if mediated through digital tools due to the impersonal and at-your-fingertips nature of these technologies which makes them more suitable to maintain anonymity and avoid the psychological stress induced by face-to-face encounters. Evidence from developmental psychiatry suggests that this increased patient empowerment may ignite a virtuous circle in which patients are incentivized to take higher responsibility for taking care of their own mental health development, which is an important step in the treatment of mental illness. Patient empowerment is also promoted through the positive effect on health literacy that digital tools are likely to exert. As young patients have the opportunity to monitor their mental health continuously and autonomously, they can gain exploratory knowledge about their conditions, thereby improving their understanding of their own mental illness. Finally, the prospect of reducing stigma may create not only a direct benefit for the technology users but also a positive externality for mental health patients in general.

Despite these prospective benefits, digital mental health tools also appear to raise technical, scientific, ethical, and regulatory challenges. Proactively addressing these challenges is paramount to ensure ethical development in the digital mental health arena and increase the chances that the promissory outlook described above will materialize. Our findings reveal that many young people use digital technologies to access information about their mental health. Although the increased accessibility of such information is beneficial, it should also be viewed with caution. If the information they receive is not reliable and scientifically vetted, it may lack validity and thereby tamper both health outcomes and patient trust in mental health services. If digital mental health tools lack validity, they may provide incorrect advice. As a consequence, patients may not seek the right help they need (40). The risk of suboptimal efficacy and insufficient clinical validation has already been observed in areas of digital mental health such as direct-to-consumer neurotechnology for mental well-being (14) and intelligent assistive technology for people with dementia and/or age-related cognitive decline (15, 16). If digital mental health tools cannot ensure efficacy and reliability, it is unlikely they can improve health outcomes and reduce the burden of mental illness. In addition, the increasing reliance on machine learning and other AI models for prediction and human-machine interaction needs to be vetted to ensure scientific validity, reliability, and transparency. Although machine learning algorithms appeared to improve existing decision support tools, their usefulness in the clinical setting was deemed limited by an ongoing lack of information on model building and uncertain accuracy (54–56). Further, it has been noted that there has yet to be clinical evaluation of predictive technologies for digital health interventions (57).

Our findings indicate that digital mental health tools may help ensure a greater degree of anonymity compared to face-to-face consultations and thereby reduce stigma. However, this beneficial potential can only materialize if digital mental health technologies ensure high standards of data security and information privacy. Privacy breaches have already been observed in several digital mental tools such as mobile health apps, wearables, consumer neurotechnologies and assistive devices for psychogeriatric care (15, 58, 59). These privacy weaknesses include illicit access by third parties to confidential patient-related information, cybercrime and accidental data leakage. Data security and privacy weaknesses are likely to have a negative snowball effect on patient trust and the doctor-patient relationship (19, 26, 28, 32, 34, 41). Also, they are expected to decrease the acceptability of digital mental health technologies (40, 43) among younger people.

With regard to patient autonomy, digital mental health tools appear to be a double-edge sword. On the one hand, they hold potential for increasing the autonomy and sense of empowerment of young adults. On the other hand, they also raise the risk of diminishing patient autonomy by increasing the risk of digital addiction and manipulation (60, 61).

Overall, our findings suggest that digital mental health technologies can improve the quality of mental healthcare provision and the quality of life of younger patients. At the same time, they indicate that technology is not a panacea for all mental health problems affecting young people's mental health and that ethical issues must be proactively addressed. When navigating these issues, special attention should be devoted to the specific needs and wishes of each patient and age subgroup. We recommend that future research on this topic should focus on specific subpopulations such as low-to-moderate and subsyndromal cases. As young people constitute a broad and heterogenous age group, it is important to look at target subpopulations within this cluster and identify the necessary codesign requirements for these end users.

These findings may provide a useful informative basis for public decision-making on digital mental health for younger people. Our thematic analysis supports the view that leveraging both technical and normative interventions holds potential for maximizing the benefits of digital health technologies while minimizing the risks. In particular, technical solutions such as cryptography and secure multi-party computation can raise the bar of device and software security, hence increase the protection of patient-generated data and protect patient privacy. In parallel, ethical guidelines for digital mental health systems can help improve the safety and efficacy of these systems and establish best practices for ethical design, responsible innovation and successful clinical implementation. However, improving safety and efficacy standards cannot be achieved exclusively through guidelines and other soft-law or hard-law interventions, but also requires a paradigm shift of the digital mental technology industry toward a culture of stewardship and responsible innovation. The Organisation for Economic Co-operation and Development's (OECD) Recommendation on Responsible Innovation in Neurotechnology (2019) offers an internationally accepted framework for promoting responsible innovation in this field. These principles and standards, however, have to be adequately implemented into product design, development, and experimentation. In particular, enhancing clinical validation standards can improve effectiveness and safety only if the holistic well-being of the patient is put at the forefront of the digital mental health enterprise and novel technologies are developed and assessed using patient-centered and participatory approaches to technology development. Our findings suggest that a shift toward patient-centered design is particularly necessary for digital mental health technologies for younger people as the needs of younger people are typically under-addressed (62). Furthermore, developing comprehensive implementation concepts appears necessary to avoid translational bottlenecks and ensure the successful translation of digital mental health technologies from the designing laboratories to personalized solutions for end-users. In order to increase the accessibility of digital mental health services, including in rural areas and among disadvantaged socio-economic segments, stakeholders should explore interventions that could lower the costs of sufficiently validated digital mental health services for individual users. Our findings reveal a number of proposed strategies to achieve this aim such as promoting the adoption of open-source hardware and software as well as adopting cost reimbursement plans by healthcare providers. The recurrent focus on fairness and access equality suggests that avoiding the exacerbation of socio-economic inequalities via digital tools is a paramount requirement for the ethically aligned deployment of these technologies. Rather than aggravating the digital divide, digital mental health tools should expand young people's access to mental health services by enabling a more widespread delivery of technology-mediated care in rural areas, among economically disadvantaged groups and among patient groups who—due to the nature of their pathology—would particularly benefit from reducing the frequency of face-to-face encounters. In order to ensure the successful adoption of these technologies among children and adolescents, policy makers should consider collaborating with educational institutions and seek the integration of these technological resources into the school setting. School-based mental health practice holds promise in meeting unmet mental health needs of children and adolescents by expanding access to quality mental health care for hard-to-reach populations (55). The recurrent emphasis put by the literature on educational resources suggests that incorporating digital mental health tools into school-based mental health practice could improve the delivery of mental health services to children, expand the resources available to educators and health providers, and monitor the effectiveness of digital mental health interventions in a systematic way. Collaborative activities involving educators, healthcare providers, technology developers and end-users are highly needed to ensure the effective and responsible deployment of digital mental health technologies for the benefit of younger people.

## Author Contributions

BW contributed to the review protocol, performed the review, data analysis, and co-wrote the manuscript. CL contributed to the data analysis and co-wrote the manuscript. MI conceived of the study, developed the research protocol, contributed to data analysis, and co-wrote the manuscript. All authors contributed to the article and approved the submitted version.

## Conflict of Interest

The authors declare that the research was conducted in the absence of any commercial or financial relationships that could be construed as a potential conflict of interest.

## Publisher's Note

All claims expressed in this article are solely those of the authors and do not necessarily represent those of their affiliated organizations, or those of the publisher, the editors and the reviewers. Any product that may be evaluated in this article, or claim that may be made by its manufacturer, is not guaranteed or endorsed by the publisher.
