# Supplementary material for: Digital Mental Health for Young People: A Scoping Review of Ethical Promises and Challenges
Source: Front Digit Health. 2021 Sep 6;3:697072. doi: 10.3389/fdgth.2021.697072 (PMC8521997; doi:10.3389/fdgth.2021.697072)
Supplement: Supplementary file 1 [file Table_1.DOCX]

Annex 1: Inclusion and exclusion criteria

|  | Inclusion criteria | Exclusion criteria |
| --- | --- | --- |
| Search string | Articles searched based on the search string* in the following databases (Pubmed, Scopus, PsychInfo, IEEExplore, World of Science, ACM Digital Library) |  |
| Retrieved article type | - Original peer-review article in scientific journal | - Proceedings to conferences, workshops or symposia - Study protocols - Letters to the editor - Viewpoint articles - Book chapters - Blog articles |
| Target group | Children and young adults under the age of 25 (average age of participants) | - Adults above the age of 25 |
| Technology type | - Digital systems - Directly design for target group and/or used by them - With potential application to brain and mental health across domains such as: prevention, diagnostics, assistive technology, monitoring, and therapeutics. | - Non-digital systems - Technologies designed for different target groups - With no reported application to brain and mental health |
| Collected data | - Quantitative measurements - Digitalised qualitative assessments (e.g., self-reports by target group or by attending physicians such as psychiatrists) |  |
| Ethical content of paper | - Discussion of ethical considerations is prominent in the article - Discussion of ethical considerations is not the core topic of the article but a dominant theme in the article | - Ethical considerations not addressed in the article - Ethical considerations mentioned tangentially in the article but not elaborated |

- *Search string: “("big data" OR "Artificial Intelligence" OR "digital phenotyping" OR "digital mental health" OR "digital biomarkers" OR "mental health apps" OR "digital sensors" OR "digital mental health technologies" OR "health related Apps" OR "mobile Health" OR eHealth OR smartphones OR wearables OR "Holter monitoring" OR "social media" OR "Digital Media" OR "Forums and Blogs") AND(ethics[Title/Abstract] OR bioethics[Title/Abstract] OR "bioethical issues"[Title/Abstract] OR "ethical issues"[Title/Abstract] OR "ethical analysis"[Title/Abstract] OR "ethical review"[Title/Abstract]) AND ("mental health" OR "mental wellbeing" OR "emotional health" OR "emotional wellbeing") AND ("young adult" OR young OR adolescent OR child OR teenager OR youth).
